# Increased biological relevance of transcriptome analyses in human skeletal muscle using a model-specific pipeline

**DOI:** 10.1186/s12859-020-03866-y

**Published:** 2020-11-30

**Authors:** Yusuf Khan, Daniel Hammarström, Bent R. Rønnestad, Stian Ellefsen, Rafi Ahmad

**Affiliations:** 1 Department of Biotechnology, Inland Norway University of Applied Sciences, Holsetgata 22, 2317 Hamar, Norway; 2 Section for Health and Exercise Physiology, Department of Public Health and Sport Sciences, Inland Norway University of Applied Sciences, Lillehammer, Norway; 3 Swedish School of Sport and Health Sciences, Stockholm, Sweden; 4 Innlandet Hospital Trust, Lillehammer, Norway; 5 Faculty of Health Sciences, Institute of Clinical Medicine, UiT - The Arctic University of Norway, Hansine Hansens veg 18, 9019 Tromsø, Norway

**Keywords:** RNA-seq, Skeletal muscle, Bioinformatics pipeline, Normalization

## Abstract

**Background:**

Human skeletal muscle responds to weight-bearing exercise with significant inter-individual differences. Investigation of transcriptome responses could improve our understanding of this variation. However, this requires bioinformatic pipelines to be established and evaluated in study-specific contexts. Skeletal muscle subjected to mechanical stress, such as through resistance training (RT), accumulates RNA due to increased ribosomal biogenesis. When a fixed amount of total-RNA is used for RNA-seq library preparations, mRNA counts are thus assessed in different amounts of tissue, potentially invalidating subsequent conclusions. The purpose of this study was to establish a bioinformatic pipeline specific for analysis of RNA-seq data from skeletal muscles, to explore the effects of different normalization strategies and to identify genes responding to RT in a volume-dependent manner (moderate vs. low volume). To this end, we analyzed RNA-seq data derived from a twelve-week RT intervention, wherein 25 participants performed both low- and moderate-volume leg RT, allocated to the two legs in a randomized manner. Bilateral muscle biopsies were sampled from *m. vastus lateralis* before and after the intervention, as well as before and after the fifth training session (Week 2).

**Result:**

Bioinformatic tools were selected based on read quality, observed gene counts, methodological variation between paired observations, and correlations between mRNA abundance and protein expression of myosin heavy chain family proteins. Different normalization strategies were compared to account for global changes in RNA to tissue ratio. After accounting for the amounts of muscle tissue used in library preparation, global mRNA expression increased by 43–53%. At Week 2, this was accompanied by dose-dependent increases for 21 genes in rested-state muscle, most of which were related to the extracellular matrix. In contrast, at Week 12, no readily explainable dose-dependencies were observed. Instead, traditional normalization and non-normalized models resulted in counterintuitive reverse dose-dependency for many genes. Overall, training led to robust transcriptome changes, with the number of differentially expressed genes ranging from 603 to 5110, varying with time point and normalization strategy.

**Conclusion:**

Optimized selection of bioinformatic tools increases the biological relevance of transcriptome analyses from resistance-trained skeletal muscle. Moreover, normalization procedures need to account for global changes in rRNA and mRNA abundance.

## Background

Skeletal muscle is a highly adaptable tissue that responds to environmental stress by altering growth rates and differentiation processes. During resistance training, signaling cascades that stimulate muscle plasticity are triggered. Upon repeated exposures, this facilitates growth and a phenotypic shift in a metabolically active direction [1], with the opposite happening during inactivity [2]. Despite this generalized view, muscle responsiveness and plasticity vary, both in response to different resistance-training protocols [3] and, perhaps more importantly, between individuals [4, 5]. Selected individuals show a near-complete absence of muscle growth after prolonged resistance training, which markedly reduces the beneficial outcomes of such interventions for muscle function and overall health [4, 5]. Currently, little is known about the etiology of this variation. However, it is usually associated with phenotypic traits of skeletal muscle [6–8], which implies interactions with environmental factors, genetics, epigenetics, and composites of the intra physiological milieu [9, 10]. This multifaceted origin makes the training-response-spectrum difficult to study directly, with each of the underlying factors offering limited explanatory value alone [11]. Instead, a more indirect approach is necessary, whereby the combined effects of the factors are targeted by studying global patterns of mRNA, protein expression, and skeletal muscle biology.

Previous studies have investigated transcriptome responses to acute resistance exercise [12–14] and chronic resistance training [12, 13, 15–18], as well as described associations between transcriptome characteristics and degrees of muscle growth [18, 19], and function [20, 21]. Whereas these studies have merited interesting findings, they lack clear coherences in terms of differential expression events, even for classical exercise-inducible genes such as PGC1$\alpha$ [22]. This lack of clear coherence is potentially due to a combination of issues such as differences in study design and methods for synthesis and analysis of transcriptome data. First, biologically founded variability can be attributed to differences in exercise protocols (e.g., differences in exercise-volume or intensity). This makes it difficult to discern a general transcriptome exercise response, as training variables are not standardized between studies. Biological heterogeneity is also caused by differences between research participants, affecting signal-to-noise ratios and making it difficult to discern the effects of single independent factors such as training variables. Design stage decisions such as the use of within-participant designs [3, 23] are likely to reduce this variation and to provide transcriptome data with increased biological meaningfulness. Second, technical variability can be attributed to decisions made during the bioinformatical treatment of data. As described by Concea et al*.* [24], there is no optimal pipeline for sequencing technology as new tools keep evolving and emerging, different tools should be explored to an optimum pipeline for the specific type of data. To exploit the potential of any study design, there is a need for identifying an appropriate pipeline for transcriptome analyses to ensure a biologically valid interpretation of data. This entails identifying potential violations of common assumptions caused by the experimental model at hand, relating to, for example, data normalization [25, 26].

For transcriptome data to provide adequate biological information about a given experimental set-up, numerous bioinformatic steps need to be adopted in a customized manner [24, 27]. Of these steps, data normalization is particularly decisive [26], as it aims to transform naïve transcript counts into biologically meaningful results. This essentially means expressing them as *per*-cell abundances [28]. For most experimental models, this is equivalent to providing transcript-to-total RNA ratios, given the fulfillment of the assumption that total RNA levels remain stable between conditions on a per-unit-cell or per-unit-tissue basis [28]. In cell models that exhibit high degrees of plasticity, gene expression events result in increased amounts of total RNA and mRNA transcripts per cell [29], specifically violating the assumption that most genes are not differentially expressed [25, 28]. We are not aware of any study that has addressed the need to account for such perspectives during transcriptome analyses of skeletal muscle subjected to mechanical stress, such as resistance training. Indeed, this assumption can be expected to be violated, as total RNA content increases markedly on a per-unit-weight basis [3], with potential global changes also occurring for the mRNA pool, though this remains unknown. The extent to which total RNA, and therefore ribosomal RNA, increases, coincides with the increase in muscle mass [3, 7], underlining its importance for cellular growth but also its inevitable presence as a potential confounding factor in RNA sequencing experiments.

In this study, we aimed to (1) establish a bioinformatic pipeline specific for analysis of RNA-seq data from skeletal muscles, (2) explore the effects of using different normalization strategies for analyzing skeletal muscle tissue subjected to resistance training, and (3) identify genes responding to moderate versus low resistance exercise volume, while simultaneously identifying genes whose expression changes with time. To achieve these aims, we utilized RNA-seq data generated from a within-participant study, comparing the effects of low and moderate resistance training volume, as previously described [3]. Also, myosin heavy chain protein expression, quantified using immunohistochemistry, was used to validate RNA quantification tools.

## Results

**Fig. 1:**
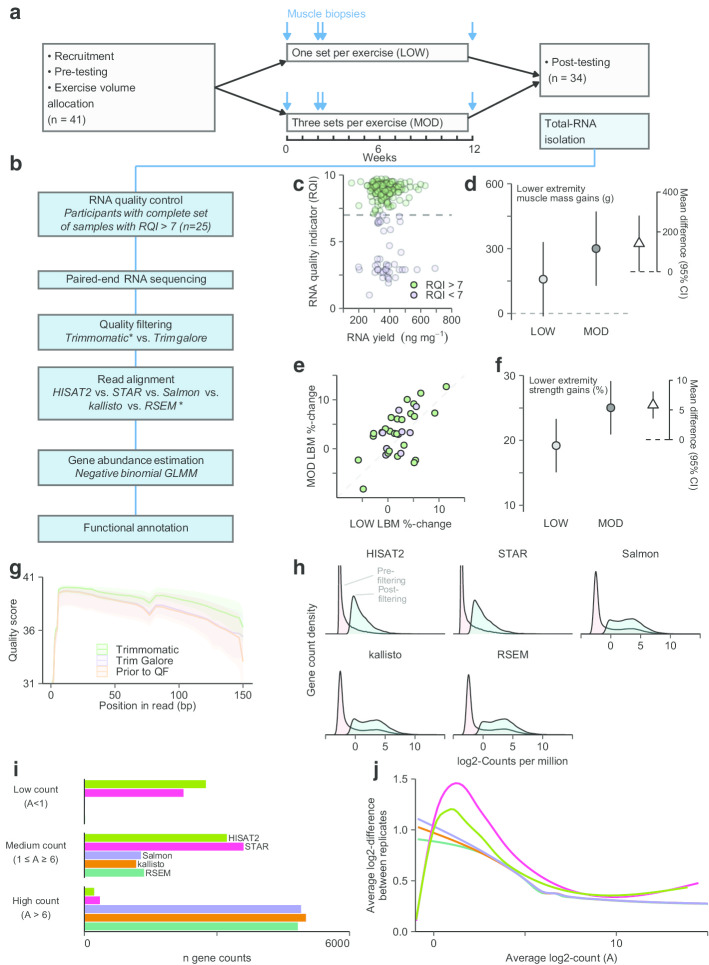
Study overview and RNA-seq analysis pipeline. Forty-one participants performed twelve weeks of resistance training with low- (one set per exercise, LOW) and moderate-volume (three sets per exercise, MOD) in a contralateral manner (2–3 sessions week-1) (**a**). Pre- and post-training testing included strength and muscle lean-mass assessments. Muscle biopsies were collected from *m. vastus lateralis* at four time-points, prior to and after the intervention (Week 0 and 12) and before and after the fifth training session (Week 2). Biopsies from participants who completed > 85% of prescribed sessions were used for RNA extraction (*n* = 34; A). RNA quality was assessed (**b**), and participants with RNA quality indicator (RQI) scores > 7 were submitted for RNA-seq (*n* = 25). RNA quality was not associated with muscle tissue weight (**c**), and participants included in RNA-seq experiments did not differ from excluded in terms of limb lean-mass gains (**d**). Higher training volume led to greater gains in limb lean mass (**e**) and strength (**f**) in the lower extremities (*n* = 25). RNA-seq data were quality filtered using trimgalore and trimmomatic and reads were compared to unfiltered reads (**g**). Read alignment was performed using five tools of which RSEM, kallisto, and Salmon showed greater fractions of genes with robust expression after removing low-abundance genes (expression filtering; H) compared to HISAT2 and STAR. RSEM, kallisto and Salmon also showed less Log2-differences between biological replicates in a subset of genes with known robust expression (see text for details, **i**)

For the RNA-seq analyses presented here, a subset of participants was selected based on RNA quality measurements from a previously reported study comparing the functional and biological efficacy of low- and moderate-volume resistance training [3] (Fig. 1a). Twenty-five participants (out of 34) had a full set of RNA-samples with RNA quality indicator (RQI) scores ≥ $7$, which were subjected to bioinformatic data analysis (Fig. 1b). RQI scores were not associated with RNA yield (Fig. 1c). In these participants, twelve weeks of training with moderate volume led to greater increases in limb lean-mass than low volume (3.5% vs. 2.0%, pre-training MOD mean (SD) 8.9 (2.2), to post-training 9.2 (2.3) kg; pre-training LOW 8.9 (2.2), to post-training 9.0 (2.2) kg, Fig. 1d), corresponding well with MRI-based muscle cross-sectional area data from the full data set [3]. Similar responses were seen in the excluded participants (Fig. 1d). This coincided with greater strength gains (~ 25% vs. ~ 19%, Fig. 1f), which also agrees with observations made in the full cohort, accompanied by greater changes in muscle fiber proportions (type IIX fibers $\downarrow$) [3].

## Bioinformatic pipeline for analysis of RNA-seq data from skeletal muscles

**Fig. 2:**
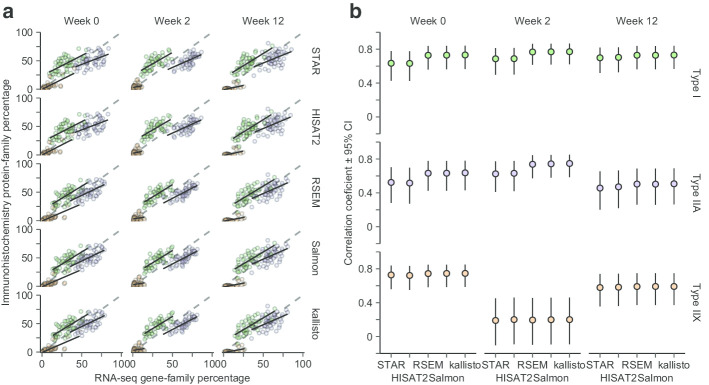
Correlations between myosin heavy chain mRNA and protein abundance. mRNA abundances of myosin-heavy chains in *m. vastus lateralis* estimated using RSEM, kallisto, and Salmon showed stronger correlations with immunohistochemistry-determined protein expression than HISAT2 and STAR (**a**, **b**). mRNA and protein abundances of *MYH7*/Type I, *MYH2*/Type IIA, and *MYH1*/Type IIX were calculated as percentages of overall myosin-heavy chain mRNA and protein expression, analyses unbiased by normalization [34, 46]

To select the most appropriate tools for bioinformatic analyses, we first compared quality filtering using Trimmomatic and Trim Galore, both of which are commonly used [30, 31]. Quality scores were generally better with Trimmomatic (Fig. 1g). Trim Galore did not improve scores over non-filtered data (Fig. 1g). Filtered reads were then aligned to the human genome using three alignment-based methods (including HISAT2, STAR, RSEM, all used together with Bowtie 2) and two non-alignment-based methods (kallisto and Salmon). RSEM, Salmon and kallisto all showed similar characteristics in terms of gene counts, resulting in the expected bimodal distribution of counts and a larger subset of detected genes after expression filtering compared to STAR and HISAT2 (Fig. 1h). For a selection of genes with known robust expression across tissues [32], Salmon, kallisto, and RSEM resulted in higher proportions of genes with high count numbers (Fig. 1i). RSEM was found to be associated with lower technical variation than Salmon, kallisto, HISAT2, and STAR expressed as log-differences in the expression of these genes between bilateral biopsies sampled prior to the intervention (Fig. 1j). For HISAT2 and STAR, this distorted correlations between RNA-seq based myosin heavy chain family mRNA and myosin heavy chain protein profiles (Fig. 2a and b), with the latter identified in Hammarström et al*.* [3], which are established hallmarks of skeletal muscle biology [33–35]. Overall, RSEM, kallisto, and Salmon thus displayed superior technical performance than HISAT2 and STAR, resulting in data with lower degrees of technical variation and higher degrees of biological validity. RSEM displayed slightly lower average variation between paired samples and was thus chosen for downstream analyses.

## Effects of normalization strategies on transcriptomic data analysis from skeletal muscle under hypertrophic stress

**Fig. 3:**
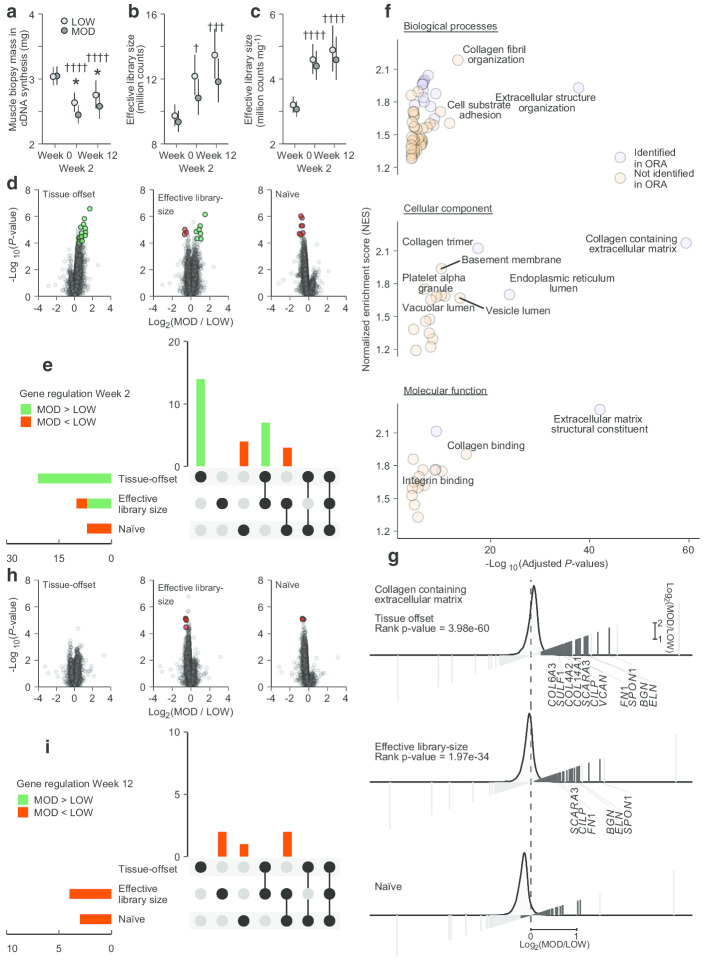
Global mRNA expression and transcriptome profiles in response to low and moderate volume resistance training. The amounts of muscle tissue used during cDNA synthesis varied over the course of the study and between volume conditions (**a** low-volume, LOW; moderate-volume, MOD). Library sizes increased during the course of the intervention, with a tendency towards a greater increase in the low-volume condition (**b**). Difference in library sizes between volume conditions when expressed per-unit tissue weight were diminished, though increases from baseline were maintained (**c**). The tissue offset-normalized model identified 21 genes with higher expression in the moderate volume condition (**d**, **e**), ten of which was shared with the effective library-size normalized model at week 2 (**e**), and none of which was shared with the naïve model. No volume-dependent differences were found at Week 12 using the tissue-offset model. At this time point, library-size and naïve models both showed a marked skew towards augmented expression in the low-volume condition. At Week 2, functional annotation identified gene sets relating to extracellular matrix in response to higher training volume (tissue-offset model, orange and purple circles, **f**), all of which were more highly expressed in MOD, indicated by the positive enrichment score. Orange circles denote gene sets that were identified from rank-based enrichment tests based on the full data set. Purple circles denote gene categories that were also identified using over-representation analysis (ORA). Normalization strategies had global effects on enrichment analyses using rank tests, assessed using fold-changes and minimum significant differences scores (not shown), illustrated with the tissue-offset model leading to marked increases in genes associated with the “Collagen containing extracellular matrix” gene set (**g**) as well as a shift in the full distribution of Log2 fold-changes between volume conditions towards MOD (shown as density curves). Black bars represent genes that belong to the gene set identified as enriched (**g**). Genes symbols indicate genes identified as differentially expressed in each normalization scenario

In the selected participants, similarly to what has been reported in the full cohort [3], resistance training led to an increase in total RNA per-unit tissue weight that was larger in response to moderate- versus low-volume training (Week 2, low 15% vs. moderate-volume 24%, mean difference 7.7%, 95% CI [1.1, 14.8]; Week 12, low 15% vs. moderate-volume 24%, mean difference 7.7%, 95% CI [1.1, 14.8]). As equal amounts of total RNA were used for preparing RNA-seq libraries, the amounts of muscle tissue used for library preparations decreased from baseline to Week 2 and 12 in both legs (low volume, − 13% and − 9%; moderate volume − 20% and − 15%). This decrease was subsequently more pronounced in the moderate volume condition, resulting in lower amounts of tissue used in cDNA synthesis (− 7.1%, 95% CI [− 12.9, − 1.0]; − 6.3%, [− 11.8, − 0.4]; Fig. 3a). Despite the utilization of less muscle tissue during library preparations in the trained state, effective library sizes increased compared to baseline levels (low volume, 25%, and 38% at Week 2 and 12, respectively; moderate volume, 16%, and 26%; Fig. 3b). Initially, this increase was numerically less pronounced in the moderate volume condition (− 11%, [− 22, 1.7]; − 12%, [− 24, 2.2]; Fig. 3b), but after normalization to tissue weight, the two training modalities resulted in similar increases in effective library size (low volume, 43%, and 53% at Week 2 and 12, respectively; moderate volume, 43%, and 49%; Fig. 3c). Overall, this suggests marked increases in global mRNA expression in response to both low- and moderate-volume resistance training.

## Identification of genes responding to moderate, compared to low exercise volume

During subsequent identification of differentially expressed (DE) genes in response to low- and moderate-volume resistance training, three normalization models were used and compared. The first model contained effective library sizes as a covariate, as previously suggested [36], while also containing tissue weight as an offset to account for amounts of tissue used during RNA-seq library preparation (tissue-offset model). The second model contained effective library sizes as a covariate only (library-size normalization), thus representing an effort to compare expression levels across training modalities while accounting for technical variation during library preparation [25, 36]. The third model was a non-normalized model (naïve model, included for comparison) (Table 1).

**Table 1:** Participant characteristics

			Mean	SD
Female	n = 11	Age (years)	22.6	0.9
		Body mass (kg)	166.2	6.2
		Stature (cm)	61.5	7.4
		Lean mass (%)	63.7	5.6
		Fat mass (%)	32.2	5.7
Male	n = 14	Age (years)	23.9	4.2
		Body mass (kg)	183.7	5.6
		Stature (cm)	77.4	10.4
		Lean mass (%)	75.4	5.5
		Fat mass (%)	20.1	5.7

Values from pre-intervention assessments. Relative lean and fat mass from whole-body data

At Week 2, 21 genes were identified as DE between low and moderate volume using the tissue-offset model, with all genes showing higher expression in the moderate volume condition (Fig. 3d; rested-state biopsies sampled after four training sessions). Similarly, 10 and seven genes were identified as DE between volume conditions using the library-size model and the naïve model, respectively (Fig. 3d and e). For the library-size model, seven of these DE genes showed higher expression in the moderate-volume condition, overlapping completely with genes found using the tissue-offset model (Fig. 3e). For the naïve model, the seven DE genes all showed decreased expression in the moderate volume condition, with 3 genes still overlapping with the library-size model, thus resulting in a contra-intuitive decrease in expression. Using tissue-offset model-derived estimates for functional analyses (Rank-based enrichment tests of minimum significant differences, MSD), revealed enrichment of genes associated with extracellular matrix (ECM) gene ontology (GO) sets (Fig. 3f, Table 2). The top-ranked GO terms were also identified by over-representation tests (ORA) using DE genes (Fig. 3f, Table 2, detailed table in Additional files 1 and 2). Using library-size model estimates similar top-ranked GO sets were identified as with the tissue-offset model albeit with lower levels of significance and lower degrees of agreement between methods (Table 2). The naïve model generally identified GO sets with negative enrichment scores indicating gene sets with lower expression in moderate volume compared to low volume, with a weak agreement between enrichment methods (Table 2). The analytical consequences of using the different normalization strategies were particularly apparent in comparisons of rank metrics, such as fold-changes. Importantly, this analytical approach uses the entire gene set to identify enriched gene sets, rather than being confined to DE genes. After controlling for amounts of tissue used during preparation of RNA-seq libraries, the distribution of Log2 differences between volume conditions shifted markedly in favor of higher training volume (Fig. 3g), and robust gene sets appeared with higher expression in the moderate volume condition, such as genes belonging to the Collagen-containing ECM GO set (Fig. 3g). Accordingly, the number of DE genes identified to this GO set was highest using the tissue-offset model (*n* = 11), followed by the library-size model (*n* = 6), with no genes identified using the naïve model (Fig. 3g).

**Table 2:** Functional annotation analysis comparing moderate- and low-volume training

Comparison	Normalization model	Gene ontology category	ID	Description	Rank *P*-value^a^	Gene-set enrichment analysis (GSEA)		ORA *P*-value^c^
						GSEA *P*-value^b^	NES	
Week 2 MOD versus LOW	Tissue offset	Biological process	GO:0043062	Extracellular structure organization	6.19e−39	6.22e−24	1.93	4.70e−06
			GO:0030199	Collagen fibril organization	1.46e−14	4.72e−11	2.22	NA
		Cellular component	GO:0062023	Collagen containing extracellular matrix	1.39e−60	7.90e−44	2.17	6.63e−12
			GO:0031012	Extracellular matrix	1.01e−58	6.10e−44	2.08	2.88e−11
			GO:0005788	Endoplasmic reticulum lumen	3.58e−25	7.82e−11	1.70	7.80e−07
			GO:0005581	Collagen trimer	9.38e−19	7.20e−12	2.14	3.89e−05
			GO:0031983	Vesicle lumen	4.15e−15	1.52e−10	1.67	NA
		Molecular function	GO:0005201	Extracellular matrix structural constituent	2.23e−43	3.67e−29	2.28	2.86e−11
			GO:0005198	Structural molecule activity	9.12e−31	3.39e−14	1.58	NA
			GO:0005518	Collagen binding	1.49e−16	5.96e−08	1.97	NA
	Effective library size	Biological process	GO:0043062	Extracellular structure organization	7.50e−21	2.68e−27	2.63	NA
			GO:0030199	Collagen fibril organization	1.56e−12	3.25e−08	2.46	NA
		Cellular component	GO:0062023	Collagen containing extracellular matrix	6.85e−35	4.30e−45	2.89	7.93e−08
			GO:0031012	Extracellular matrix	1.77e−32	3.26e−41	2.73	1.29e−07
			GO:0005788	Endoplasmic reticulum lumen	3.16e−14	1.56e−13	2.28	NA
			GO:0005581	Collagen trimer	1.88e−13	7.84e−11	2.50	NA
		Molecular function	GO:0005201	Extracellular matrix structural constituent	2.67e−31	5.53e−26	2.83	1.67e−07
			GO:0005198	Structural molecule activity	1.21e−18	7.39e−21	2.22	NA
			GO:0005518	Collagen binding	2.24e−10	1.83e−08	2.37	NA
			GO:0030020	Extracellular matrix structural constituent conferring tensile strength	8.51e−09	2.52e−06	2.26	NA
	Naïve	Biological process	GO:0006397	mRNA processing	1.76e−17	5.03e−04	− 1.48	NA
			GO:0008380	RNA splicing	3.19e−17	0.002	− 1.47	NA
			GO:0000375	RNA splicing via transesterification reactions	3.41e−15	0.005	− 1.49	NA
			GO:1903311	Regulation of mRNA metabolic process	7.48e−10	0.003	− 1.51	NA
			GO:0050684	Regulation of mRNA processing	1.18e−07	0.015	− 1.64	NA
			GO:0043484	Regulation of RNA splicing	1.76e−07	0.049	− 1.57	NA
			GO:0048024	Regulation of mRNA splicing via spliceosome	3.48e−07	0.009	− 1.75	NA
			GO:0000380	Alternative mRNA splicing via spliceosome	8.32e−07	0.027	− 1.74	NA
		Cellular component	GO:0005681	Spliceosomal complex	2.65e−11	0.007	− 1.56	NA
			GO:0016607	Nuclear speck	3.10e−08	0.010	− 1.40	NA
Week 12 MOD versus LOW	Tissue offset	Biological process	GO:0010498	Proteasomal protein catabolic process	0.046	0.685	1.00	NA
			GO:0006401	RNA catabolic process	0.046	0.737	− 0.87	NA
			GO:0006397	mRNA processing	0.046	0.904	0.88	NA
			GO:0000209	Protein polyubiquitination	0.046	0.579	1.05	NA
		Molecular function	GO:0003729	mRNA binding	0.003	0.844	0.99	NA
			GO:0019783	Ubiquitin like protein specific protease activity	0.021	0.286	1.37	NA
			GO:0019787	Ubiquitin like protein transferase activity	0.021	0.796	1.01	NA
			GO:0008234	Cysteine type peptidase activity	0.021	0.247	1.40	NA
			GO:0016874	Ligase activity	0.039	0.691	1.10	NA
			GO:0003730	mRNA 3 utr binding	0.043	0.775	1.01	NA
	Effective library size	Biological process	GO:0006613	Cotranslational protein targeting to membrane	1.18e−36	1.29e−07	− 2.11	NA
			GO:0072599	Establishment of protein localization to endoplasmic reticulum	3.52e−36	6.44e−09	− 2.12	NA
			GO:0070972	Protein localization to endoplasmic reticulum	1.52e−33	2.30e−08	− 2.04	NA
			GO:0019080	Viral gene expression	1.14e−30	1.75e−07	− 1.86	NA
		Cellular component	GO:0005840	Ribosome	6.28e−42	6.81e−12	− 2.01	NA
			GO:0044391	Ribosomal subunit	6.74e−42	4.05e−12	− 2.09	NA
			GO:0022626	Cytosolic ribosome	3.40e−36	2.36e−09	− 2.15	NA
			GO:0098798	Mitochondrial protein complex	1.20e−35	7.52e−10	− 1.89	NA
			GO:0019866	Organelle inner membrane	2.88e−35	7.52e−10	− 1.67	NA
		Molecular function	GO:0003735	Structural constituent of ribosome	7.79e−43	7.81e−12	− 2.13	NA
	Naïve	Biological process	GO:0006613	Cotranslational protein targeting to membrane	7.73e−30	3.48e−08	− 2.14	NA
			GO:0072599	Establishment of protein localization to endoplasmic reticulum	7.73e−30	1.65e−08	− 2.10	NA
			GO:0070972	Protein localization to endoplasmic reticulum	7.58e−28	6.14e−07	− 1.92	NA
		Cellular component	GO:0019866	Organelle inner membrane	4.52e−43	3.70e−11	− 1.68	NA
			GO:0098798	Mitochondrial protein complex	2.31e−40	8.47e−10	− 1.83	NA
			GO:0005840	Ribosome	4.35e−39	1.75e−10	− 1.91	NA
			GO:0044391	Ribosomal subunit	5.29e−39	2.47e−11	− 2.03	NA
			GO:0098800	Inner mitochondrial membrane protein complex	1.13e−30	6.41e−09	− 2.06	NA
			GO:0022626	Cytosolic ribosome	2.07e−30	1.75e−10	− 2.23	NA
		Molecular function	GO:0003735	Structural constituent of ribosome	2.12e−39	3.20e−12	− 2.17	NA

^a^Rank-based enrichment test based on minimum significant difference identifies gene-sets that are over-represented among top-ranked genes without a directional hypothesis

^b^Gene-set enrichment analysis (GSEA) tests for over-representation in among top and bottom genes based on Log_2_ fold-changes in comparing moderate- (MOD) versus low-volume (LOW) conditions. Positive normalized enrichment scores (NES) indicates genes with higher expression in MOD compared to LOW, negative NES indicates genes with higher expression in LOW compared to MOD

^c^Over-representation tests based on differentially expressed genes. *P*-values are adjusted for FDR

At Week 12, no genes were identified as differentially expressed between resistance training with low and moderate volume using the tissue-offset model (Fig. 3h; rested-state biopsies sampled after finalization of the intervention). In contrast, a small number of genes were identified as DE between volume conditions using library-size and naïve models (n = 4 and n = 3, respectively; Fig. 3h and i), with all genes showing lower expression in the moderate volume condition (Fig. 3h and i). Of these genes, two were shared between models (Fig. 3i). Using tissue-offset model-derived estimates for functional annotation analyses, revealed no consensus GO sets, using either of the two enrichment approaches (Table 2). In contrast, functional annotation based on estimates from the library-size and naïve model revealed GO terms related to cellular respiration and protein translation with enrichment scores (NES) indicating higher expression of genes in the low-volume condition (Table 2). No DE genes contributed to ORA-identified GO terms among the top-ranked GO terms from the library-size model. In the naïve model, genes related to cellular respiration were identified as DE with subsequent contribution in ORA-identified GO terms (Table 2).

## Identification of genes with altered expression over time (0, 2 and 12 weeks)

**Fig. 4:**
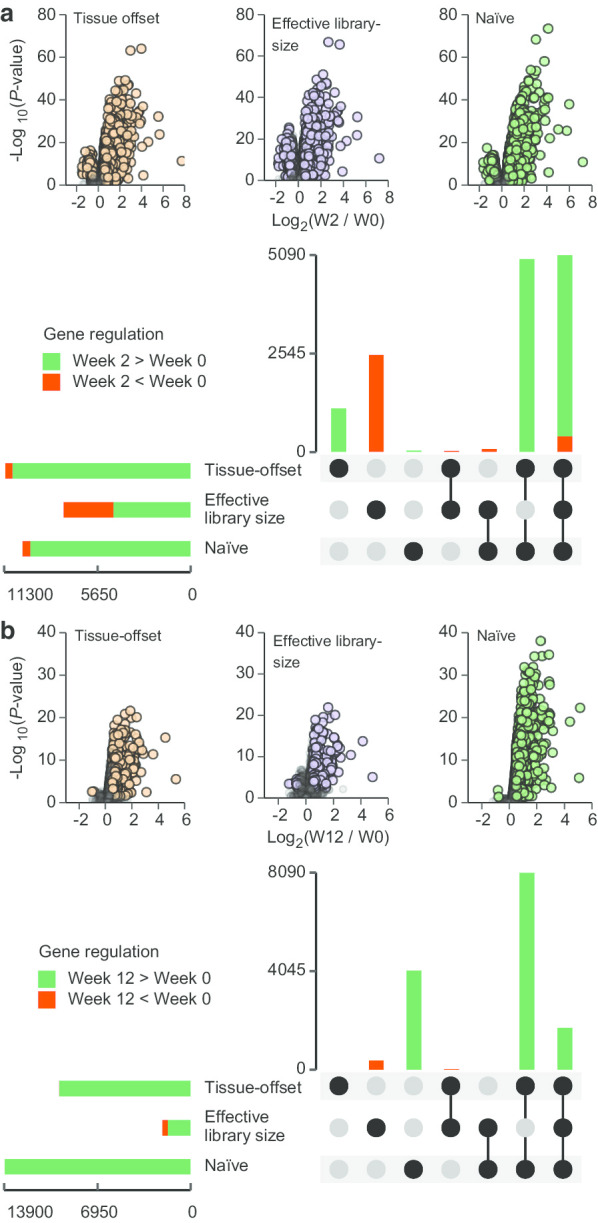
Comparing the effects of resistance training per se on transcriptome profiles using different normalization models. Volcano plot identifies differentially expressed genes at Week 2 (**a**) and Week 12 (**b**) (adjusted P-values < 0.05 and Log2 fold-changes >|0.5|, filled circles). Bar-plots shows the total number of DE genes (horizontal bars) and sets exclusively found in each model or shared among models (vertical bars). The majority of differentially expressed genes were identified by all three normalization models, though the effective library-size model identified a larger number of genes with decreased expression (**a**)

At Week 2 and 12 (rested-state biopsies), we also investigated the overall effects of resistance training on transcriptome profiles: i.e. the time effect, assessed by combining data from the two training modalities. At Week 2, resistance training led to increased expression for 3923, 1609, and 3875 genes and decreased expression of 77, 289, and 100 genes using the tissue-offset, the library-size, and the naïve model, respectively (Fig. 4a). The majority of these DE genes were found in the intersection between all models (Fig. 4a lower panel). At Week 12, resistance training led to increased expression of 1733, 584, and 5108 genes and decreased expression of 2, 19, and 2 genes using the tissue-offset, the library-size, and the naïve model, respectively (Fig. 4b). Here, the majority number of DE genes were found in the intersection between the tissue-offset model and the naïve model (Fig. 4b lower panel). At both Week 2 and 12 (and using any normalization model), functional analyses of DE genes revealed enrichment of GO terms associated with ECM structure, organization, and synthesis, as well as stress responses (Table 3).

**Table 3:** Functional annotation analysis of time-dependent effects of resistance training

Comparison	Normalization model	Gene ontology category	ID	Description	Rank P-value	Gene-set enrichment analysis (GSEA)		ORA P-value
						GSEA P-value	NES	
Week 2	Tissue offset	Biological process	GO:0043062	Extracellular structure organization	7.28e−41	5.92e−25	1.96	9.04e−28
			GO:0006954	Inflammatory response	1.28e−30	5.92e−25	1.79	9.04e−28
			GO:0002274	Myeloid leukocyte activation	4.48e−24	3.87e−14	1.59	2.40e−16
			GO:0050900	Leukocyte migration	4.79e−22	4.62e−18	1.82	2.20e−17
			GO:0002444	Myeloid leukocyte mediated immunity	3.46e−20	4.71e−12	1.57	8.98e−15
		Cellular component	GO:0031012	Extracellular matrix	5.85e−70	5.45e−51	2.17	3.58e−52
			GO:0062023	Collagen containing extracellular matrix	1.19e−68	3.15e−47	2.20	5.88e−53
			GO:0005788	Endoplasmic reticulum lumen	6.10e−24	2.71e−12	1.75	5.21e−17
			GO:0005581	Collagen trimer	1.29e−21	9.26e−14	2.24	1.29e−10
		Molecular function	GO:0005201	Extracellular matrix structural constituent	1.70e−40	3.56e−24	2.23	8.04e−30
	Effective library size	Biological process	GO:0043062	Extracellular structure organization	1.28e−34	6.94e−23	2.03	4.27e−29
			GO:0006954	Inflammatory response	3.11e−26	3.15e−21	1.82	9.15e−28
			GO:0050900	Leukocyte migration	5.50e−18	1.58e−13	1.83	8.59e−15
			GO:0030199	Collagen fibril organization	1.01e−17	2.75e−11	2.28	2.49e−14
			GO:0042330	Taxis	5.63e−17	1.74e−12	1.69	2.15e−16
		Cellular component	GO:0031012	Extracellular matrix	2.64e−63	7.23e−43	2.17	1.48e−53
			GO:0062023	Collagen containing extracellular matrix	2.38e−61	4.14e−38	2.20	4.43e−52
			GO:0005581	Collagen trimer	3.52e−20	8.15e−12	2.23	1.18e−14
			GO:0005788	Endoplasmic reticulum lumen	8.79e−18	2.51e−12	1.87	1.50e−10
		Molecular function	GO:0005201	Extracellular matrix structural constituent	9.00e−38	6.36e−18	2.18	2.82e−28
	Naïve	Biological process	GO:0043062	Extracellular structure organization	1.38e−40	2.25e−23	1.93	3.36e−26
			GO:0006954	Inflammatory response	1.30e−31	2.61e−26	1.78	5.70e−28
			GO:0002274	Myeloid leukocyte activation	3.44e−23	4.99e−14	1.57	6.42e−16
			GO:0050900	Leukocyte migration	7.26e−23	8.32e−17	1.79	7.78e−20
			GO:0042330	Taxis	2.04e−19	4.63e−18	1.69	5.41e−17
		Cellular component	GO:0031012	Extracellular matrix	4.84e−71	2.20e−54	2.15	7.81e−53
			GO:0062023	Collagen containing extracellular matrix	9.15e−70	7.75e−49	2.19	1.01e−53
			GO:0005788	Endoplasmic reticulum lumen	4.50e−24	2.56e−12	1.72	4.68e−18
			GO:0005581	Collagen trimer	8.56e−22	1.06e−13	2.20	2.17e−11
		Molecular function	GO:0005201	Extracellular matrix structural constituent	1.98e−40	4.68e−25	2.20	6.38e−29
Week 12	Tissue offset	Biological process	GO:0043062	Extracellular structure organization	1.86e−49	2.01e−28	2.22	1.09e−37
			GO:0001501	Skeletal system development	7.13e−21	6.49e−14	1.77	1.09e−16
			GO:0030199	Collagen fibril organization	2.46e−20	4.83e−12	2.48	1.29e−15
		Cellular component	GO:0031012	Extracellular matrix	8.63e−72	3.48e−58	2.47	2.23e−69
			GO:0062023	Collagen containing extracellular matrix	4.96e−69	4.97e−54	2.52	8.25e−67
			GO:0005581	Collagen trimer	3.65e−25	1.27e−19	2.64	2.40e−25
			GO:0005788	Endoplasmic reticulum lumen	8.11e−20	1.85e−10	1.84	1.87e−13
		Molecular function	GO:0005201	Extracellular matrix structural constituent	2.46e−47	1.34e−34	2.63	4.24e−46
			GO:0005539	Glycosaminoglycan binding	1.48e−20	3.73e−15	2.15	2.01e−17
			GO:0008201	Heparin binding	5.72e−19	1.03e−15	2.25	9.82e−16
	Effective library size	Biological process	GO:0043062	Extracellular structure organization	1.79e−44	3.76e−21	1.90	8.48e−33
			GO:0030199	Collagen fibril organization	4.77e−19	3.05e−08	2.10	2.69e−10
		Cellular component	GO:0031012	Extracellular matrix	6.91e−67	6.04e−41	2.03	2.38e−61
			GO:0062023	Collagen containing extracellular matrix	1.43e−63	2.57e−38	2.07	2.59e−56
			GO:0005581	Collagen trimer	1.74e−24	6.46e−13	2.17	1.11e−26
			GO:0005788	Endoplasmic reticulum lumen	1.45e−17	8.84e−09	1.66	1.58e−13
		Molecular function	GO:0005201	Extracellular matrix structural constituent	3.54e−45	1.48e−21	2.13	4.31e−42
			GO:0005198	Structural molecule activity	3.18e−20	2.05e−14	1.61	NA
			GO:0005539	Glycosaminoglycan binding	1.36e−18	2.43e−12	1.90	1.05e−15
			GO:0008201	Heparin binding	2.49e−18	7.91e−11	1.95	6.20e−16
	Naïve	Biological process	GO:0043062	Extracellular structure organization	2.90e−52	1.61e−39	2.94	1.06e−28
			GO:0001501	Skeletal system development	7.74e−23	3.14e−18	2.21	1.05e−11
			GO:0030199	Collagen fibril organization	2.99e−21	1.57e−15	3.14	5.82e−08
		Cellular component	GO:0031012	Extracellular matrix	1.49e−81	7.09e−79	3.31	2.66e−44
			GO:0062023	Collagen containing extracellular matrix	4.03e−79	1.04e−69	3.39	4.01e−46
			GO:0005581	Collagen trimer	5.29e−27	6.03e−27	3.39	4.83e−12
			GO:0005788	Endoplasmic reticulum lumen	8.14e−24	5.17e−16	2.37	1.45e−14
		Molecular function	GO:0005201	Extracellular matrix structural constituent	1.22e−50	7.93e−48	3.59	6.42e−28
			GO:0005539	Glycosaminoglycan binding	7.61e−24	1.07e−19	2.78	6.84e−13
			GO:0008201	Heparin binding	1.83e−21	1.44e−18	2.90	1.97e−09

^a^Rank-based enrichment test based on minimum significant difference identifies gene−sets that are over-represented among top-ranked genes without a directional hypothesis

^b^Gene−set enrichment analysis (GSEA) tests for over-representation in among top and bottom genes based on Log_2_ fold-changes in comparing time−points (Week 2 vs. Week 0 and Week 12 vs. Week 0). Positive normalized enrichment scores (NES) indicate genes with higher expression in Week 2/12 compared to Week 0; negative NES indicates genes with higher expression in Week 0 compared to Week 2/12

^c^Over-representation tests based on differentially expressed genes. *P*-values are adjusted for FDR

## Effects of acute exercise on transcriptome profiles (pre- to post-exercise in Week 2)

**Fig. 5:**
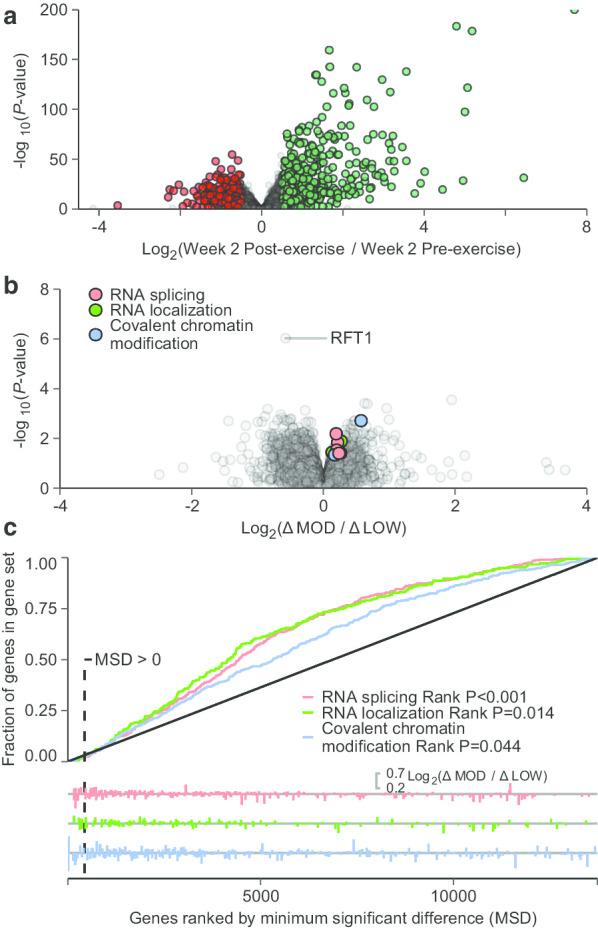
Comparing the effects of an acute bout of resistance training with low and moderate volume on transcriptome profiles in muscle biopsies. Overall, an acute bout of resistance training led to large-scale alterations in gene expression (volume-conditions combined) (**a**). Comparing differentially expressed events between volume conditions (low-volume, LOW; moderate-volume, MOD) identified one gene with volume-dependent changes in expression (RFT1, **b**). Three gene ontology categories were identified as significantly enriched using a rank-based test (minimum significant difference, MSD as the rank metric) as well as containing genes with unadjusted *P*-values < 0.05 (MSD > 0). Traces from the rank test are displayed in **c**

At Week 2, we also investigated the effects of acute bouts of resistance exercise with low and moderate volume on transcriptome profiles. As we did not expect changes in the total RNA-to-muscle mass ratio in this short time span [37] and rather fluid shifts [38] may have affected tissue weight and hence downstream analyses in an undesirable manner, transcriptome analyses were performed using the library-size model. First, we performed an analysis of the effects of resistance training per se on transcriptome profiles (combining data from the two training modalities). These analyses identified 1736 DE genes after acute resistance exercise, 707 of which showed increased expression and 1029 of which showed decreased expression (Fig. 5a). Genes that showed increased expression were generally associated with stress-related GO terms, including immune response (Table 4). Genes that showed decreased expression were associated with ECM-related GO terms (Table 4), contrasting observations made in rested-state biopsies at Week 2 and 12 (Table 3; detailed table in Additional files 1 and 2). We then compared the effects of low and moderate-volume conditions. These analyses identified one single DE gene (RFT1, Fig. 5b), which decreased to a greater extent in the moderate-volume condition. Despite this, rank-based enrichment tests with MSD identified five GO terms with significant enrichment. Among these five categories, three had genes with MSD > 0 (RNA splicing, RNA localization, and Covalent chromatin modification), indicating that the lower bound of 95% CI did not overlap no-change. However, as differences between volume conditions were both negative and positive, as indicated by the rug-plot in Fig. 5c, these categories were not identified in gene-set enrichment analysis based on fold-changes. Overall, these analyses do not provide evidence for pronounced volume-dependent regulation of mRNA expression in the acute recovery phase after resistance exercise (1-h).

**Table 4:** Functional annotation analysis of time−dependent effects of acute resistance exercise

Gene ontology category	ID	Description	Rank P-value	Gene−set enrichment analysis (GSEA)		ORA P-value
				GSEA P-value	NES	
Biological process	GO:0050900	Leukocyte migration	2.31e−16	0.010	1.58	1.04e−04
	GO:0009617	Response to bacterium	6.61e−14	1.42e−04	1.81	8.54e−06
	GO:0060326	Cell chemotaxis	6.69e−14	0.019	1.61	1.15e−04
	GO:0006954	Inflammatory response	2.08e−12	0.019	1.45	0.048
	GO:0002237	Response to molecule of bacterial origin	2.08e−12	4.61e−05	2.04	2.29e−05
	GO:0042330	Taxis	1.11e−11	9.24e−04	1.68	3.05e−05
	GO:0007159	Leukocyte cell cell adhesion	1.17e−11	0.003	1.78	4.02e−04
	GO:0030595	Leukocyte chemotaxis	8.29e−11	0.038	1.54	0.004
	GO:0048514	Blood vessel morphogenesis	8.72e−11	1.20e−05	1.89	1.78e−11
	GO:0042110	T cell activation	1.25e−09	0.022	1.46	6.84e−04
Cellular component	GO:0072562	Blood microparticle	3.23e−04	0.726	1.20	NA
	GO:0098589	Membrane region	0.004	0.162	1.39	NA
	GO:0042581	Specific granule	0.009	0.644	1.18	NA
	GO:0070820	Tertiary granule	0.055	0.444	1.28	NA
	GO:0005667	Transcription factor complex	0.070	1.93e−04	1.88	NA
	GO:0000932	P body	0.082	0.243	1.49	0.049
	GO:1903293	Phosphatase complex	0.095	0.012	1.97	0.049
	GO:0030055	Cell substrate junction	0.110	0.033	1.49	NA
	GO:0051233	Spindle midzone	0.116	0.647	1.29	NA
	GO:1904724	Tertiary granule lumen	0.180	0.091	1.81	NA
Molecular function	GO:0001216	DNA binding transcription activator activity	8.87e−15	1.50e−14	2.56	NA
	GO:0035326	Cis regulatory region binding	7.65e−10	3.00e−15	2.38	0.035
	GO:0030545	Receptor regulator activity	3.34e−07	0.004	1.75	0.003
	GO:0005125	Cytokine activity	8.04e−07	0.002	2.00	0.004
	GO:0001217	DNA binding transcription repressor activity	2.71e−06	6.07e−04	1.89	NA
	GO:0001968	Fibronectin binding	3.90e−05	0.411	1.30	0.024
	GO:0001664	G protein coupled receptor binding	3.90e−05	0.078	1.52	0.047
	GO:0008083	Growth factor activity	4.22e−05	0.001	2.03	0.004
	GO:0005126	Cytokine receptor binding	2.11e−04	9.14e−04	1.94	0.047
	GO:0140272	Exogenous protein binding	3.80e−04	0.198	1.54	NA
Biological process	GO:0043062	Extracellular structure organization	1.21e−11	0.006	− 1.57	0.046
	GO:0001501	Skeletal system development	6.32e−09	0.057	− 1.32	0.008
	GO:0072676	Lymphocyte migration	3.54e−07	0.126	− 1.42	NA
	GO:0032963	Collagen metabolic process	1.43e−06	0.032	− 1.61	NA
	GO:0002697	Regulation of immune effector process	1.60e−06	0.115	− 1.31	NA
	GO:0002250	Adaptive immune response	6.08e−06	0.023	− 1.43	NA
	GO:0070661	Leukocyte proliferation	6.23e−06	0.055	− 1.40	NA
	GO:0060348	Bone development	7.92e−06	0.031	− 1.49	NA
	GO:0042098	T cell proliferation	1.68e−05	0.127	− 1.34	NA
	GO:0033627	Cell adhesion mediated by integrin	1.69e−05	0.288	− 1.27	0.007
Cellular component	GO:0031012	Extracellular matrix	8.06e−15	7.19e−11	− 2.12	NA
	GO:0062023	Collagen containing extracellular matrix	8.74e−12	3.62e−13	− 2.29	NA
	GO:0005581	Collagen trimer	3.23e−04	7.19e−11	− 2.56	NA
	GO:0009897	ExteRNAl side of plasma membrane	0.001	0.023	− 1.57	NA
	GO:0005788	Endoplasmic reticulum lumen	0.008	0.003	− 1.69	NA
	GO:0098552	Side of membrane	0.012	0.460	− 1.18	NA
	GO:0043235	Receptor complex	0.014	0.210	− 1.29	NA
	GO:0043202	Lysosomal lumen	0.032	0.002	− 1.91	NA
	GO:0035579	Specific granule membrane	0.065	0.839	− 1.09	NA
	GO:0098802	Plasma membrane signaling receptor complex	0.092	0.263	− 1.37	NA
Molecular function	GO:0005201	Extracellular matrix structural constituent	2.40e−07	6.68e−10	− 2.38	NA
	GO:0005539	Glycosaminoglycan binding	1.38e−06	0.251	− 1.27	0.048
	GO:0008201	Heparin binding	7.60e−06	0.224	− 1.33	NA
	GO:0005178	Integrin binding	7.04e−04	0.103	− 1.42	NA
	GO:1901681	Sulfur compound binding	8.19e−04	0.098	− 1.35	NA
	GO:0030246	Carbohydrate binding	8.22e−04	0.230	− 1.27	NA
	GO:0005518	Collagen binding	0.004	0.050	− 1.66	NA
	GO:0043394	Proteoglycan binding	0.010	0.185	− 1.53	NA
	GO:0050840	Extracellular matrix binding	0.011	0.351	− 1.27	NA
	GO:0019838	Growth factor binding	0.016	0.195	− 1.35	NA

^a^Rank-based enrichment test based on minimum significant difference identifies gene−sets that are over-represented among top-ranked genes without a directional hypothesis

^b^Gene−set enrichment analysis (GSEA) tests for over-representation in among top and bottom genes based on Log_2_ fold-changes in comparing time−points (Post- vs. Pre−exercise). Positive normalized enrichment scores (NES) indicate genes with higher expression Post- compared to Pre−exercise, negative NES indicates genes with higher expression Pre− compared to Post-exercise

^c^Over-representation tests based on differentially expressed genes. *P*-values are adjusted for FDR

## Discussion

In the present study, we used a within-participant model to study the effects of low and moderate resistance training volumes on transcriptome responses. For these analyses, we used a subset of muscle biopsy material from a previously reported study [3]. Training volume led to robust increases in muscle strength and limb lean mass, resembling observations made in the full study cohort [3], and previous studies [39]. Despite these benefits, few differences were detected in transcriptome profiles between volume conditions, with the most prominent exception being a selection of genes involved in extracellular matrix organization and biology in the early stages of resistance training. We have shown that disclosure of these differences was made possible by our systematic selection of bioinformatic tools.

## Identification of a model-specific bioinformatics pipeline

The continued development of bioinformatic tools for RNA-seq analyses require continuous optimization of analytic pipelines to any specific study conditions [24, 40]. To this end, we first sought to select a suitable read-trimming method. This is necessary to provide high-quality downstream alignment and k-mer search in reads [30]. Two commonly used algorithms were compared [30, 31]. Trimmomatic provided data with higher quality than Trimgalore (Fig. 1G), which did not improve quality scores compared to non-filtered data. Second, we compared five mapping tools for performing transcript quantification of trimmed reads: two genome-based mapping tools (STAR [41] and HISAT2 [42]) and three transcript based mapping tools (RSEM [43], kallisto [44] and Salmon [45]). Transcript-based mapping tools resulted in stronger correlations between mRNA and protein profiles, measured as relationships between myosin heavy chain mRNA profiles and protein abundances in rested state biopsies [3] (Fig. 2a), which is known to correlate in resting human skeletal muscle [33–35]. This comparison was performed using gene-/protein-family normalization [34, 46], allowing the deduction of mRNA-to-protein relationships without the need for other normalization assumptions. Notably, a marked skew was observed in the relationship between *MYH1* proportions and its corresponding Type IIX fiber following the initial part of the training intervention. This coincided with robust changes in *MYH1* expression, as is typically seen in response to mechanical loading in such short time frames [47]. Furthermore, the transcript-based mapping tools (RSEM, kallisto, and Salmon) resulted in transcriptome profiles with an expected bimodal distribution of counts and a larger subset of detected genes compared to genome-based tools (Fig. 1h). They were also associated with the less technical variation, evident as lower Log2-fold differences in expression for a selection of highly expressed genes between the two legs at baseline [23], assuming minimal biological variation between such paired samples. In these analyses, RSEM displayed slightly lower average variation between paired samples, thus outperforming kallisto and Salmon (Fig. 1j).

## Comparison of normalization strategies

Transcriptome analyses often rely on the assumption that gene expression is counted and compared between conditions on a per-cell level [28]. This is implicitly assumed to be equivalent to measuring transcriptome data as ratios between mRNA and total RNA, as the input in sequencing or hybridization experiments usually is total RNA [13, 15, 48].

In the current study population, we previously showed that total RNA increases per-unit-muscle tissue in a volume-dependent manner following initiation of resistance training [3]. Consequently, the preparation of cDNA libraries for RNA-seq experiments was unavoidably based on different amounts of tissue, as fixed amounts of total RNA (1000 ng) were used for this purpose. If unaccounted for, this would lead to a comparison of transcriptome data originating from different amounts of muscle tissue for the two volume conditions. We show that this leads to a contra-intuitive larger increase in global transcript counts in the low-volume condition compared to the moderate-volume condition (though without reaching statistical significance). In contrast, after adjusting for the difference in amounts of muscle tissue fed into RNA-seq experiments (i.e., tissue-offset normalization), this apparent difference in average library size disappeared. Overall, we thus observed an increase in global mRNA expression per-unit-muscle weight (43–53%) in response to resistance training that did not depend on training volume, contrasting the observed volume-dependency of total RNA expression [3]. This global change in mRNA expression was associated with substantial alterations in the expression of a multitude of genes, with as many as 26 and 12% of the total read-count pool showing increased expression using the tissue-offset model at Week 2 and Week 12, respectively. These genes were associated with biological processes such as ECM synthesis and organization corroborating with previous studies [13, 18, 22].

As the volume-dependent changes in muscle growth and total RNA levels in *m. vastus lateralis* arguably will affect downstream bioinformatics analyses and identification of DE genes, we aimed to compare three different normalization strategies during the subsequent analyses: tissue-offset, library-size, and naïve. At Weeks 2 and 12, the different normalization strategies resulted in marked shifts in global mRNA responses between the two volume conditions, having pronounced effects on identification of GO terms during enrichment analyses (Fig. 3g). In general, tissue-offset normalization (providing mRNA expression per-mg-muscle weight) resulted in a global shift in mRNA responses towards moderate volume. At Week 2, this was evident as more robust increases in mRNA expression in response to higher training volume for most genes, contrasting findings in library-size and naïve analyses. At Week 12, this was evident as a counterbalancing of mRNA expression profiles, with tissue-offset providing rather normally distributed responses to the two volume conditions, contrasting the skew towards larger mRNA expression in response to low training volume in library-size and naïve analyses. The utilization of generalized linear mixed models (GLMM) allowed convenient comparisons of normalization models, which could then be fitted using in the same statistical framework, as previously suggested [36]. GLMM also allowed the incorporation of random effects into the model to account for the repeated measures design. Although there are approaches to account for correlated observations in commonly used RNA-seq modeling frameworks [49], GLMMs provides a more robust and potentially more powerful framework for dealing with correlated data [36].

## Training volume-dependent changes in transcriptome profiles.

Using the tissue-offset model, we were able to identify genes relating to ECM functions as volume-sensitive during the early stages of resistance training. This may indicate a role for ECM remodeling in the beneficial effects of higher training volumes on muscular adaptations and strength. As such, previous research has shown that ECM remodeling is induced by exercise training, both acutely [50–53], and after prolonged endurance and resistance training [13, 18, 54, 55], at both mRNA and protein levels [13, 18, 54, 55]. However, none of these previous studies have found ECM remodeling to be differentially affected by different exercise modalities. Rather, different resistance training modalities such as low- and high-load training have been associated with similar responses, measured as collagen synthesis [52]. Importantly, there seems to be a close association between training-induced changes in abundances of ECM-related mRNAs and their respective proteins, including collagen-organization proteins [54]. This suggests that ECM remodeling is primarily controlled at the transcriptional level, which arguably increases the biological relevance of the herein presented transcriptome analyses. However, this relationship seems to involve a complex time course dependency. For example, transcriptional regulation of COL1A2 shows a considerable lag from stimuli to transcription, as shown in fibroblasts [56]. In line with this, our data on the effects of acute resistance exercise on transcriptome profiles, suggests a counterintuitive reduction in the expression of e.g., collagen mRNA immediately after exercise, as has also been found by others [18]. Indeed, enrichment analyses confirmed these negative changes in ECM-related transcripts in response to acute exercise, contrasting the effects of chronic resistance training [13, 18].

The physical properties of ECM are distorted by disuse and aging, resulting in increased stiffness and potentially decreased force transmission and muscle efficiency [57, 58]. Training-induced ECM remodeling may thus constitute an effective measure to reverse these adversities [50–53], and has been suggested to exert a protective role against injury [51]. However, available studies are ambiguous in their conclusions [52, 53], and the link between observed changes in ECM-related gene expression and muscle biology and functionality remain uncertain. Adding to this, ECM remodeling-responses to training seems to be age-dependent [50, 59], and also shows a clear dependency of time. Indeed, in the present study, the volume-associated differences in ECM-related transcriptome profiles disappeared entirely after twelve weeks of resistance training, whereby no genes were identified as showing volume-dependent responses. From a general perspective, this indicates that after prolonged training, the biological state of the muscle may have reached a new equilibrium, with low- and moderate-volume training having led to similar muscle phenotypic traits. This would imply that the benefits of higher training volume are restricted to augmented increases in muscle mass, perhaps facilitated by increases in ribosomal biogenesis [3]. Notably, this is likely to be an oversimplification. Indeed, at the protein level, in the current study, higher training volume led to a more robust phenotypic switch from type IIX $\to$ type IIA fiber also after twelve weeks of training [3]. Taken together, our data provide valuable directions for future research. It suggests that ECM remodeling is volume-dependent, at least during the initial part of a training program. This needs to be confirmed by studies in other populations, and its biological and functional significance needs clarification. Such studies should take advantage of the increased biological resolution of within-subject contralateral models.

Analyses of transcriptome responses to acute bouts of low and moderate resistance training volume revealed one single gene with volume-dependent changes in expression (RFT1). RFT1 is associated with the GO terms lipid transport, carbohydrate transport, and endoplasmic reticulum membrane. RFT1 expression has previously been shown to decrease in muscle immediately after training [22]. Although this warrants more research, one single gene arguably provides limited information. As such, rank-based enrichment tests identified three GO terms with volume-dependent changes in gene expression in the acute data set (RNA splicing, RNA localization, and covalent chromatin modification). Upon closer examination, these gene sets consisted of genes with both increased and decreased expression in the moderate- compared to the low-volume condition. Only a small fraction of these transcripts showed actual positive MSDs, indicating changes with unadjusted *P*-values < 0.05. While this enrichment analysis supports acute volume-dependent regulation of gene sets after resistance training at the selected time point of biopsy sampling (1 h after sessions), it remains plausible that such regulation would have been more pronounced at later time points.

## Conclusions

Transcriptomic analyses of skeletal muscle subjected to altered growth conditions should account for global changes in mRNA to total RNA and cell density to accurately reflect biologically meaningful events. When accounting for such aspects, ECM remodeling showed volume-dependent responses to resistance training. These recommendations could be applicable to studies of other cell types and model systems undergoing increased or arrested growth. Also, the optimized selection of bioinformatic tools increases the biological relevance of transcriptome analyses from resistance-trained skeletal muscle.

## Methods

### Participants and study overview

The full study design has been previously described in detail [3]. Thirty-four participants completed a 12-week training-intervention with legs allocated to either low- (one set per exercise) or moderate-volume (three sets per exercise) training (Fig. 1a). Muscle biopsies were obtained from each leg prior (Week 0) to and after the intervention (Week 12), as well as prior to (Week 2 Pre-ex) and 60-min after (Week 2 Post-ex) the last training session of week 2, as previously described [3]. Participants with a complete set of high-quality RNA samples (RQI $\ge$ 7, *n* = 25) were selected for RNA-seq analyses (Fig. 1b). Training-induced changes in muscle size and strength were estimated for each leg using several methods (for a complete overview, see [3]). Herein, we present dual-energy X-ray absorptiometry (DXA) measurement of lean mass for the 25 participants eligible for RNA-seq, as well as a weighted combined measure of strength (combining data from different strength tests) (Table 1).

## Training protocol

The training protocol consisted of unilateral lower body exercises (leg-press, leg-curl, and knee-extension). Each participant’s leg was randomly assigned to perform either one or three sets per exercise (low- vs. moderate-volume), ensuring within-participant comparisons. Rest periods between sets were 90–180 s. The single-set leg was always trained in the rest period between the second and third sets of the multiple-set protocol. Training protocols were performed in a progressive manner, whereby resistance was continuously adjusted to ensure that the targeted number of repetitions was reached at volatile fatigue. This was equivalent to 10 repetitions maximum (RM) in weeks one and two, followed by 8RM in weeks three to five and 6RM in weeks six to twelve. Each week consisted of either 2 or 3 training sessions. From week four, weeks with three sessions contained one session at a sub-maximal load (90% of previous session load). All sessions commenced with a standardized warm-up. After each session, participants were given a standardized milk-based drink [3].

## Muscle strength and lean mass assessments

Muscle strength was assessed twice before and once after the intervention. A detailed description of strength outcomes resulting from the study has been previously reported [3]. For the purpose of the present analyses, we present a weighted average of strength gains for the 25 participants eligible for RNA-seq, based on data from unilateral isometric and isokinetic (60°, 120° and 240° $\times$ s^−1^) knee extension, and one-repetition maximum (1RM) in unilateral knee extension and leg press, as previously reported [3]. Isometric and isokinetic strength was assessed using an individually adjusted dynamometer (Cybex 6000, Cybex International, Medway USA). 1RM was defined as the maximum load lifted through the full range of motion. From pre-intervention tests, the highest values were used for change score calculations. Limb lean-mass was assessed from full-body dual-energy X-ray absorptiometry (DXA; Lunar prodigy, GE Healthcare, Oslo, Norway) scans performed prior to and after the intervention. Limb lean-mass was derived from a segment covering the full leg from collum femoris to the distal end of the foot defined in the analysis software (enCore, GE Healthcare, Oslo, Norway).

## Muscle tissue sampling, immunohistochemistry and RNA extraction

Muscle tissue was obtained bilaterally from m. vastus lateralis using a 12-gauge needle (Universal-plus, Medax, San Possidonio, Italy) under local anesthesia (Xylocaine, 10 ${\text{mg}} \times {\text{ml}}^{ - 1}$ with adrenaline 5 μg × ml^−1^, AstraZeneca AS, Oslo, Norge). Samples were obtained from the two legs within 10 min of each other at all time-points. All rested state samples were obtained in the morning after a standardized breakfast. Resting samples obtained in Week 2 were sampled approximately 48 h after the fourth session. After the training period (Week 12), samples were obtained six days after the last training session and three days after the last strength assessment. Participants were instructed to ingest standardized meals during the 24 h leading up to the sampling event, and to refrain from strenuous physical activity during the last 48 h. Samples for immunohistochemistry (~ 15 mg) were transferred to a 4% formalin solution for fixation 24 − 72 h, before further preparation. Samples for RNA analyses (~ 25 mg) were dissected in ice-cold sterile saline solution (0.9% NaCl), blotted dry and snap-frozen using −80 °C isopentane, before storage at −80 °C until further processing.

Immunohistochemistry was utilized to quantify myosin heavy chain abundance in formalin-fixed muscle biopsy cross-sections, performed as previously described and reported [3]. Briefly, 4 µm transverse sections were incubated with (1) a primary antibody that detects all three adult myosin heavy chain isoforms but type IIX (BF-35, 5 μg × ml^−1^, Developmental Studies Hybridoma Bank, deposited by Schiaffino, S.) and (2) type I myosin (MyHCSlow, 1:4000, catalog M8421L, Sigma-Aldrich Norway AS, Oslo, Norway). Primary antibodies were visualized using BMU UltraView DAB and UltraView Red (Ventana Medical Systems, Inc. Tucson, USA). Muscle fibers were identified as either Type I (red), Type IIA (brown), Type IIX (unstained), or hybrid fibers Type IIA/IIX (light brown) (for representative images, see Fig. 3 in [3]). Hybrid fibers were analyzed as 0.5 × Type IIA and 0.5 × Type IIX.

For RNA extraction, the frozen muscle was homogenized in 1 ml of TRIzol reagent (Invitrogen, Life technologies AS, Oslo, Norway) using a bead homogenizer (Bullet Blender, Next Advanced, Averill Park, NY, USA). After phase separation, 400 μl of the aqueous phase was used in isopropanol precipitation of RNA, and after three washing steps (70% ethanol) the pellet was eluted in TE buffer. All samples showed a 260/280 ${\text{nm}}$ ratio > 1.95 assessed by a spectrophotometer (NanoDrop 2000, ThermoFisher Scientific, Oslo, Norway). RNA integrity scores (RQI) were determined by capillary electrophoresis (Experion Automated Electrophoresis Station using RNA StdSens Assay, Bio-Rad). For each participant, all samples were extracted in the same extraction session in a randomized order. Participants with complete sets of high-quality RNA samples had an average RQI score of 9.0 (0.4), [full data set, 8.1 (2.1), range 1–9.7] (Fig. 1c). Notably, to achieve accurate normalization of qPCR data (and potentially also RNA-seq data), a commercially available exogenous RNA control (λ polyA External Standard Kit, Takara Bio Inc., Shiga, Japan) was added at a fixed amount per extraction prior to homogenization (0.04 ng ml^−1^ of Trizol reagent), as previously described [3, 60]. Unfortunately, at present, we do not have access to the sequence of this spike-in, prohibiting its identification in RNA-seq data and rendering its subsequent utilization for normalization purposes difficult.

## Illumina library preparation and sequencing

For each muscle sample, mRNA sequencing libraries were prepared from the same amount of RNA (1000 ng, depending on the minimum amount available) using TruSeq Stranded Total RNA Library Prep (Illumina, San Diego, CA USA). Paired-end sequencing (150 bp) was performed using an Illumina HiSeq 3000 (Illumina) at the Norwegian Sequencing Centre.

## Bioinformatic analysis

### Pre-alignment filtering

Trim Galore (version 0.6.5, https://github.com/FelixKrueger/TrimGalore) and Trimmomatic (version 0.39) [31] were used to discard low-quality reads and trim poor-quality bases before alignment, using default settings. The quality of filtered files was calculated by FastQC (version 0.11.4) and summarized using MultiQC (version 1.8) [61].

### Read alignment

Filtered reads were aligned to the Human genome (GRCh38 release-97 downloaded from ftp.ensemble.org) using the alignment-based methods HISAT2 (version 2.1.0) [42], STAR (version 2.7.2) [41], and RSEM (version 1.3.1) [43], used together with Bowtie 2 (version 2.3.4.3) [62], and non-alignment methods including kallisto (version 0.44.0) [44] and Salmon (version 0.13.1) [45]. For HISAT2 and STAR, HTSeq was used for quantification as previously described [63]. RSEM, kallisto, and Salmon have in-built quantification functions.

### Modeling of gene counts

Gene counts were modeled using negative binomial generalized linear mixed models (GLMM), as suggested in [36], with modifications. Three model formulations were used to represent three different normalization scenarios. First, to account for fluctuations in RNA-to-tissue ratios, the amount of tissue used in cDNA synthesis was included as an offset term together with the effective library size and study conditions (time and volume condition), added as a fixed effects in the model (tissue offset model). A simplified model contained only the effective library size together with study conditions, included as fixed effects (Effective library-size model). And finally, a naïve model formulation, without any form of normalization term, was used for comparisons. For acute exercise effects (pre- to post-exercise in the last session of week 2), only the library size normalized model was used as we expected that fluid shifts [38] could influence the muscle weight measurement, and changes in total RNA were unlikely to occur in this short time span [37]. The effective library size was calculated by multiplying the total library size with the RNA composition normalization factor, calculated using the trimmed mean method [25], followed by dividing the value by the median effective library size, as suggested by Cui et al. [36]. The effect of resistance training on gene counts was assessed as (1) the effect of exercise volume and (2) the effect of time. For analyses of the effect of exercise volume, differential expression was evaluated using GLMMs containing the interaction between time and exercise volume. For analyses of the effect of time, differential expression was evaluated using GLMMs containing only the time factor, combining all data irrespective of volume condition. In all models, a single random effect was used, giving each participant an individual intercept. Models were iteratively fitted using glmmTMB [64]. Utilization of the negative binomial distribution was supported by comparing the full model with a Poisson model (not containing the dispersion term), providing likelihood-ratio tests *P*-values that were distributed primarily below 0.05 (0.37% of models showed *P* > 0.05). Heteroscedasticity was assessed using the uniformity test in the DHARMa package [65], which generally showed good agreement with model assumptions, providing *P*-values concentrated near 1 (98.51% of models showed *P* > 0.05). Genes were identified as differentially expressed when the absolute Log2 fold-change was greater than 0.5, and the adjusted *P*-value was below 5%. *P*-values were adjusted per-model coefficient to control the false discovery rate [66].

## Functional annotation

Enrichment analyses of gene ontology (GO) gene sets were performed using three approaches. First, a non-parametric rank test (described in [67] and implemented in the tmod package [68], version 0.40) was performed based on gene-specific minimum significant differences (MSD). MSD was defined as the lower limit of the 95% confidence interval (CI, based on estimated standard errors) around the Log fold-change (FC) when Log(FC) > 0 and the negative inverse of the upper 95% CI when Log(FC) < 0. This metric has been shown to have lower false-positive rates compared to other metrics applied during enrichment analyses [69]. As the MSD metric is positive when the CI does not overlap 0 and negative when overlap occurs, the rank test does not discern between up and down-regulated gene sets. A second approach, gene set enrichment analysis (GSEA) [70], was used to quantify the directional regulation of the gene set. GSEA was performed using the fgsea package [71] with Log(FC) as the gene level metric. Thirdly, over-representation analysis (ORA) was performed to assess if genes identified as differentially expressed (|Log2 fold-change|> 0.5 and adjusted *P*-values < 0.05; DE-genes) belonged to specific gene sets. ORA was performed using the enrichGO function in the clusterProfiler package [72], (version 3.16.0). GO gene sets (biological process, cellular component, and molecular function) were retrieved from the molecular signature database (version 7.1) [73].

## Statistical analysis

Descriptive data are presented as mean and standard deviation (SD). Changes in muscle strength and mass were estimated using linear mixed models on change scores with baseline values as covariates. The performance of alignment tools was assessed by comparing log-differences between biological replicates, as suggested by Teng et al. [74], with modifications. Briefly, a subset of genes previously shown to be stably expressed between tissues was selected [32], whereupon log-differences between paired biopsy samples were calculated (i.e., using biopsies collected from each of the two legs prior to the training intervention). In addition, alignment tools were assessed by comparing relationships (Pearson’s correlation coefficient) between gene family profiling of myosin heavy chains (*MYH1*, *MYH2*, and *MYH7*; muscle-specific) and the corresponding myosin heavy chain protein expression (measured using immunohistochemistry as fiber types IIX, IIA, and I). These mRNA and protein profiles were expressed as a fraction of total counts, thus removing the need for normalization of the RNA-seq data, as previously described for qPCR data [34]. Notably, these data also provided insight into the overall biological validity of the RNA-seq data, linking gene counts to protein phenotypes.

## Supplementary information

**Additional file 1:** Gene count estimates. Model (negative binomial generalized linear mixed models) based estimates of gene counts from models using different normalization strategies. ensemblid: Ensembl gene identifiers; normalization_model: Indicates normalization model: lib_size_normalized, gene counts are modeled with study conditions as fixed effects and participant id as random effects and with the addition of library size as a fixed effect. tissue_offset_lib_size_normalized, same as lib_size_normalized but with the addition of tissue weight used in library prep as an offset. The non-normalized model is not included in the data set; interaction_model: Indicates if the coefficient is estimated in a model containing the interaction between volume condition and time. If FALSE, the model only contains time as a fixed effect (in addition to any normalization, see above), coefficients should in this case be interpreted as averages over volume conditions; coefficient: Names of coefficients (fixed effects, time and volume conditions). timew2pre and timew12 indicate differences from timew0 (intercept) in rested state models. timew2post indicates differences from timew2pre (intercept) in acute exercise models. setsmultiple indicates interaction effects, the difference between setssingle (reference level) and setsmultiple; estimate: Estimates on the natural log scale; se: Standard errors (SE) on the natural log scale; zvalue: Z-values; pvalue: Un-adjusted P-values; pvalue_adjust: Adjusted P-values (FDR) per model/normalization method and coefficient.

**Additional file 2:** Functional annotation using gene ontology terms. Significance tests for functional annotation using rank-based and over-representation based analysis. ID: Gene ontology (GO) id; go_category: GO category, bp, Biological process; cc, cellular component; mf, molecular function; name: Descriptive GO name; normalization_model: Indicate normalization model used to test enrichment: lib_size_normalized, gene counts are modeled with study conditions as fixed effects and participant id as random effects and with the addition of library size as a fixed effect, tissue_offset_lib_size_normalized, same as lib_size_normalized but with the addition of tissue weight used in library prep as an offset. The non-normalized model is not included; coefficient: Names of coefficients used to test enrichment (fixed effects, time and volume conditions). timew2pre and timew12 indicates differences from timew0 (intercept) in rested state models. timew2post indicates differences from timew2pre (intercept) in acute exercise models. setsmultiple indicates interaction effects, the difference between setssingle (reference level) and setsmultiple; cerno_statistic: Test statistic from the rank-based cerno-test; cerno_auc: Area under the curve from the Cerno test (see tmod documentation for details, https://CRAN.R-project.org/package=tmod); cerno_pval: Un-adjusted P-values from cerno test, cerno_padj: Adjusted P-values (default settings in tmodCERNOtest, see tmod documentation); fgsea_pval: Un-adjusted P-values from gene set enrichment tests performed with the fgsea package; fgsea_padj: Adjusted fgsea P-values; NES: Normalized enrichment scores from gene set enrichment tests; set_size: Size of gene sets using genes expressed in the present data set; ora_geneRatio: Gene ratio in over-representation analysis of genes identified as differentially expressed; ora_padj: Adjusted p-values from over-representation analysis.

## Data Availability

Gene count estimates are available as Additional files 1 (see description below). Functional annotation using gene ontology are available as Additional files 2 (see description below). Additional datasets and code used in analysis during the current study are available from the corresponding author on reasonable request.

## Abbreviations

DXA: Dual-energy X-ray absorptiometry
ECM: Extracellular matrix
DE: Differentially expressed
MSD: Minimum significant differences
RNA-seq: Ribonucleic acid sequencing
RQI: RNA quality indicator
ORA: Over-representation analysis
